# Supplementary material for: Measuring Single-Cell Calcium Dynamics Using a Myofilament-Localized Optical Biosensor in hiPSC-CMs Derived from DCM Patients
Source: Cells. 2023 Oct 26;12(21):2526. doi: 10.3390/cells12212526 (PMC10647603; doi:10.3390/cells12212526)

cells MDPI

Supplementary Methods

S1.0 Generation of hiPSC from peripheral blood.

All work using hiPSC in this study was approved under REB# 2020-6362 from MUHC institutional review board. Blood was collected from two patients diagnosed with dilated cardiomyopathy and one healthy control volunteer with no known cardio-pulmonary disease (see **Table S2**). To generate hiPSCs from these subjects, peripheral blood mononuclear cells were reprogrammed using an episomal plasmid-based strategy. Briefly the Epi5TM Episomal iPSC Reprogramming Kit (ThermoFisher) and Neon Transfection System (Invitrogen) were used to introduce pluripotency factors Oct4, Sox2, Lin28, Klf4, and L-Myc using electroporation. Successful reprogramming was determined by attachment to the cell culture dish and confirmed by immunostaining expression of pluripotency genes. To ensure genetic integrity, hiPSCs were karyotyped using G-banding technique (Cytogenomics platform, SickKids, Toronto).

S1.1 Differentiation of hiPSCs into cardiomyocytes.

Differentiation of hiPSCs into cardiomyocytes was accomplished via the modulation of Wnt signaling using the previously published GiWi protocol with slight modifications. [35]. Briefly, on day 0 hiPSCs were treated with 12 μM CHIR99021 to activate the WNT signalling pathway. The following day, media was replaced with RPMI1640 supplemented with B27 without insulin. On day 3 the WNT signalling pathway was inhibited using 5 μM IPW2. On day 5 media was replaced with RPMI1640 supplemented with B27 without insulin and on day 7, media was replaced with RPMI1640 supplemented with B27 with insulin. The cell population was purified using 1M lactate to select for hiPSC-CMs. Successful differentiation of hiPSCs into cardiomyocytes (CMs) was confirmed through immunofluorescence and via the expression of the cardiomyocyte-specific biosensor RGECO-TnT (Figs. 6 and 7).
Experiments were conducted 28-35 days after cells began to beat to allow maturation.

S1.2 Seeding in a 96-well microwell plate.

Note: Ideally, the assay microplate should have an optical bottom compatible with fluorescent microscopy and black walls to reduce noise during measurements.

1. Coat a black optical bottom 96-well plate with 50 μL of a human plasma fibronectin dilution (1:100 in PBS). Allow to polymerize for at least 1 hour at 37°C.

2. Add 500 μL of pre-warmed Accutase solution per well and incubate at 37°C for 10-30 minutes. Check on cells periodically to see when cells detach.

3. Following incubation, add 500 μL of plating media (44 mL RPMI1640 + 5 mL KOSR + 1 mL B27 + 50 μL Y-27632ROCK inhibitor) [35]. Gently pipette up and down with a 1000 μL filter tip, ensuring all cells detach. Collect the cell suspension in a 50 mL conical tube.

4. Count the cells using trypan blue.

5. Aspirate the human plasma fibronectin coating solution and seed cells at 20,000 hiPSC-CMs per well in a 96-well plate.

   Complete the volume to 100 μL per well with plating media.

6. The next day, rinse cells 3 times with basal RPMI1640 media to remove cell debris. Refresh media with RPMI + B27. Maintain cells in RPMI1640 + B27 by changing the media every 3-4 days.

Supplemental Figure Legends

Supplemental Figure S1. Validation of hiPSCs used in this study. (A) Immunofluorescence staining of core pluripotency markers demonstrating the "stemness" of HID041004 post-reprogramming**. (B)** Karyotyping analysis of HID041004 validating normal diploid human female karyotype. **(C)** Immunofluorescence staining of core pluripotency markers demonstrating the "stemness" of HID041020 post-reprogramming**. (D)** Karyotyping analysis of HID041020 validating normal diploid human male karyotype. **(E)** Immunofluorescence staining of core pluripotency markers demonstrating the "stemness" of HID041021 post-reprogramming**. (F)** Karyotyping analysis of HID041021 validating normal diploid human female karyotype.

Supplemental Figure S2. Images of instruments used in this study. (A) Zeiss Axio Observer fully automated inverted microscope **(B)** with the temperature- and gas-controlled stage insert for 96-well plates. **(C-E)** Auxiliary components to use with the Axio Observer microscope. When initializing the system, the gas- and temperature- controller must be turned on first. Then, the Power Supply unit, SMC stage controller, and Focus Controller are turned on in this order. Finally, the X-Cite LED source is turned on and set to 20% power.

Supplemental Figure S3. Visualization of OriginPro software. User interface shown here.

Supplemental Table S1. Equipment and software used in calcium imaging and analysis.

Name	Details	Supplier
Equipment		
**Inverted microscope**	Zeiss Axio Observer	Zeiss
**Camera**	Zeiss 506 monochrome CCD	Zeiss
**Temperature and gas controller**	LCI CU-501	Live Cell Instrument
**Power supply unit**	Power Supply 232	Zeiss
**Stage controller**	SMC 2009	Zeiss
**Definite focus controller**	Focus Controller.2	Zeiss
**LED source**	X-Cite® 120 LED	Excelitas Technologies
**Filter cube**	Filter Set 14 (item no. 488014-0000-000)	Zeiss
**20x objective lens**	20x PLAN APOCHROMAT, NA 0.8	Zeiss
Software		
**Microscopy software**	Zen Blue 3.3	Zeiss
**Image visualization**	ImageJ (1.53p) with the Bio-Formats plugin	National Institutes of Health
**Transient analysis, graphing, and statistics**	OriginPro 2022 (9.9.0.225; Student version)	OriginLab

Supplemental Table S2. Detailed information on hiPSCs used in this study.

	Biological sex	Age at study entry	Disease status
**HID041004**	Female	45	Control
**HID041020**	Male	65	DCM
**HID041021**	Female	44	DCM

Supplemental Figure S1

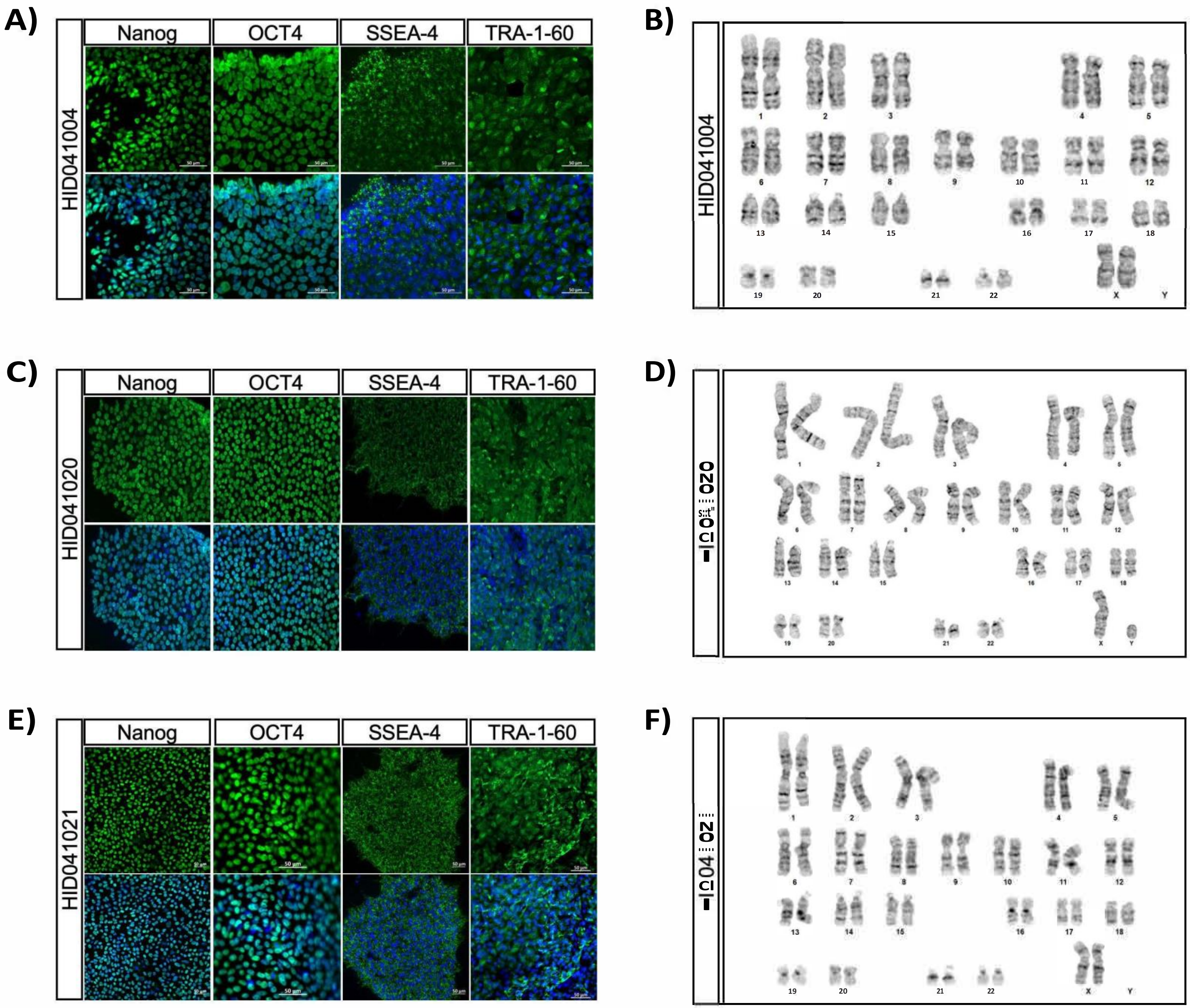

Supplemental Figure S2

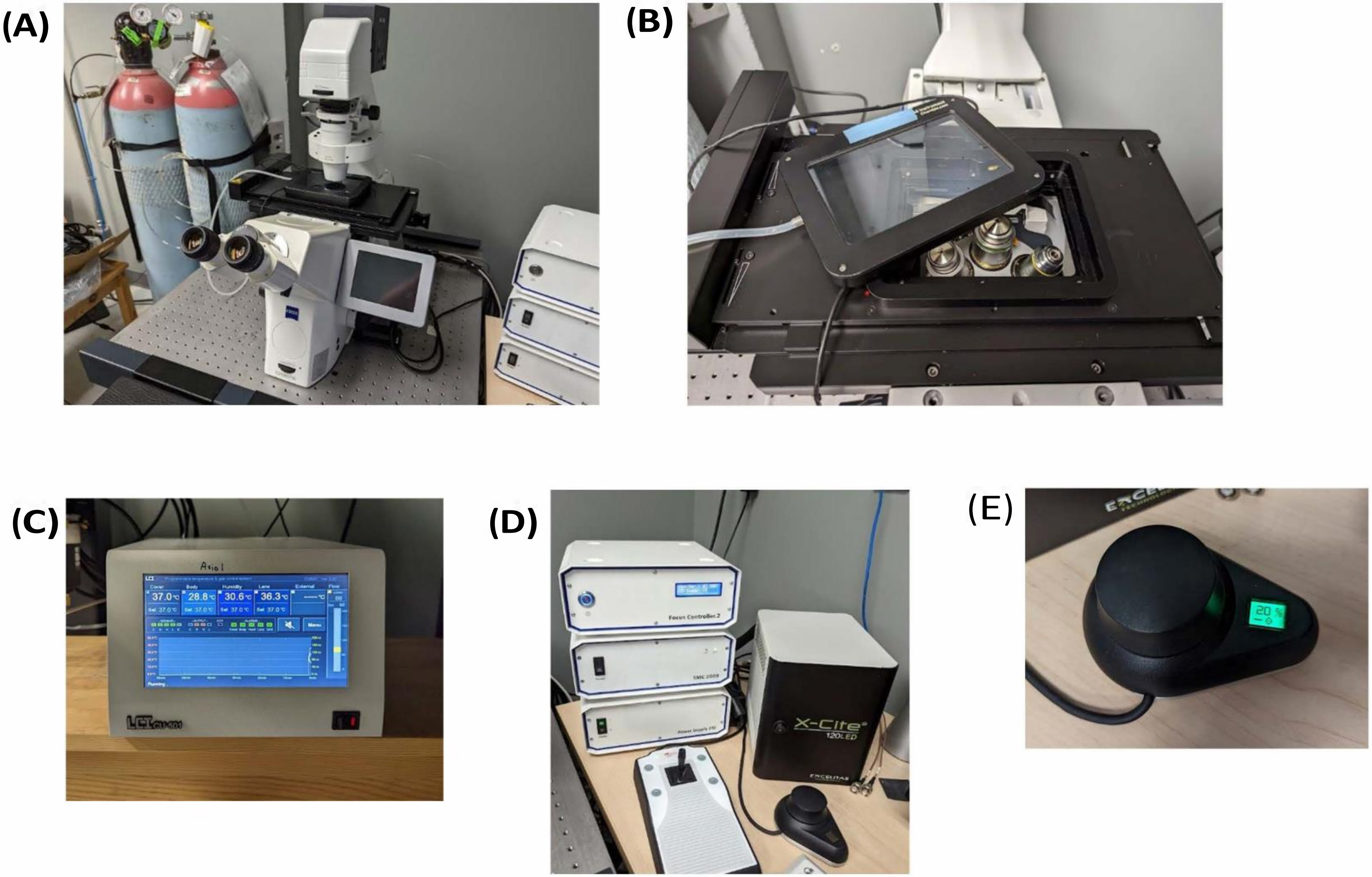

Supplemental Figure S3

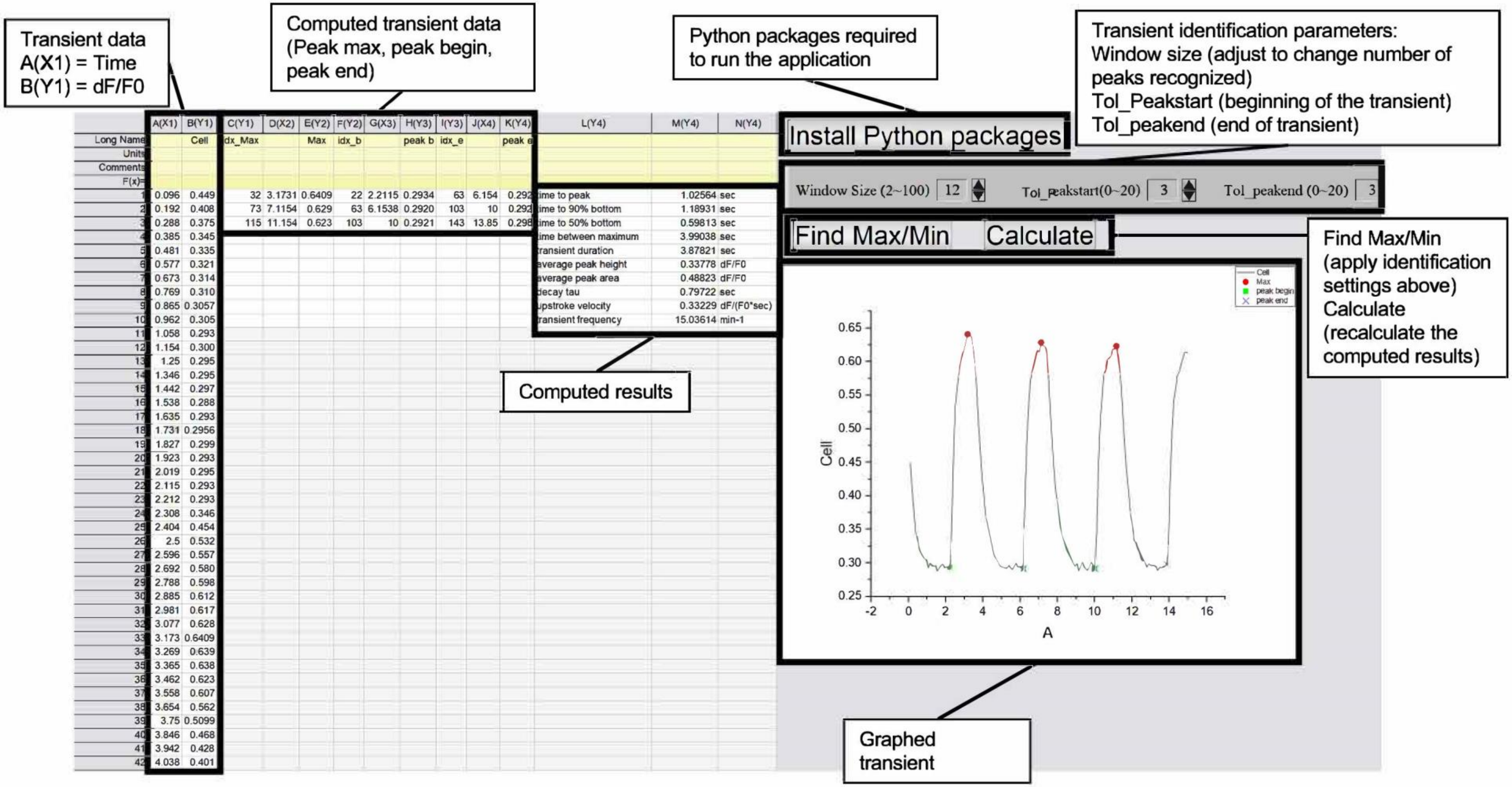

Supplement: Supplementary file 1 [file cells-12-02526-s001.zip › cells-2639473-supplementary.pdf]
